# Emerging role of mutations in epigenetic regulators including *MLL2* derived from The Cancer Genome Atlas for cervical cancer

**DOI:** 10.1186/s12885-017-3257-x

**Published:** 2017-04-08

**Authors:** Xia Li

**Affiliations:** Research Center for Biomedical Information Technology, Shenzhen Institutes of Advanced Technology, Chinese Academy of Sciences, Shenzhen, People’s Republic of China

**Keywords:** *MLL2*, Mutation, TCGA, Epigenetic regulator, Cervical cancer

## Abstract

**Background:**

Cervical cancer is the second most common cause of cancer deaths in women worldwide. The aim of this study is to exploit novel pathogenic genes in cervical carcinogenesis.

**Method:**

The somatic mutations from 194 patients with cervical cancer were obtained from the Cancer Genome Atlas (TCGA) publically accessible exome-sequencing database. We investigated mutated gene enrichment in the 12 cancer core pathways and predicted possible post-translational modifications. Additionally, we predicted the impact of mutations by scores quantifying the deleterious effects of the mutations. We also examined the immunogenicity of the mutations based on the mutant peptides’ strong binding with major histocompatibility complex class I molecules (MHC-I). The Kaplan-Meier method was used for the survival analysis.

**Results:**

We observed that the chromatin modification pathway was significantly mutated across all clinical stages. Among the mutated genes involved in this pathway, we observed that the histone modification regulators were primarily mutated. Interestingly, of the 197 mutations in the 26 epigenetic regulators in this pathway, 25 missense mutations in 13 genes were predicted in or around the phosphorylation sites. Only mutations in the histone methyltransferase *MLL2* exhibited poor survival. Compared to other mutations in *MLL2* mutant patients, we noticed that the mutational scores prioritized mutations in *MLL2*, which indicates that it is more likely to have deleterious effects to the human genome. Around half of all of the mutations were found to bind strongly to MHC-I, suggesting that patients are likely to benefit from immunotherapy.

**Conclusions:**

Our results highlight the emerging role of mutations in epigenetic regulators, particularly *MLL2,* in cervical carcinogenesis, which suggests a potential disruption of histone modifications. These data have implications for further investigation of the mechanism of epigenetic dysregulation and for treatment of cervical cancer.

**Electronic supplementary material:**

The online version of this article (doi:10.1186/s12885-017-3257-x) contains supplementary material, which is available to authorized users.

## Background

Cervical cancer remains a serious global health problem, with an estimated 528,000 new cases and 266,000 deaths in 2012 [1]. Approximately 80% of cervical cancers occur in developing countries [2]. Certain types of the human papilloma virus (HPV) infection, particularly HPV 16 and HPV 18, are the greatest risk factors for cervical cancer. Screenings, such as the Papanicolaou and HPV tests, have been largely successful in preventing cervical cancer, but it is still the second most common cause of cancer death among women worldwide, resulting in 275,000 deaths annually [3].

Not all individuals who are infected with high-risk HPVs develop genital cancer, which indicates that HPV infection is necessary but not sufficient for malignant development [4–6]. Additional genetic alterations, either independent or in conjunction with HPV infection, are required for tumor development. When cells are persistently infected with HPV, the primary viral oncoproteins E6 and E7 are reportedly involved in the disruption of many normal functions [7–10]. Consequently, these lead to an accumulation of somatic mutations. Early reports have frequently observed somatic mutations in cervical cancer [11, 12]. These genetic alterations can be equally important for cell transformation. An in-depth characterization of the underlying genetic events is important for understanding tumor progression, which can guide the development of effective targeted therapies.

The large volume of data currently generated by the TCGA provides a rich resource and a new opportunity for exploring the genetic alterations in cervical cancer. In this study, we set out to explore the pathogenic genes by investigating the somatic mutations in 194 cases of cervical cancer exome-seq data from the TCGA. The analysis showed that the chromatin modification pathway was significantly altered. Half of the epigenetic regulators involved in this pathway harbored mutations capable of disrupting the phosphorylation sites. Of all of the altered epigenetic regulators, only the histone methyltransferase *MLL2* mutations were associated with poor survival. Around half of the mutations’ peptides were predicted to be immunoreactive, which indicates that patients are likely to benefit from immunotherapy. This study highlights the emerging role of epigenetic regulators, particularly *MLL2,* and suggests potential epigenetic dysregulation, in cervical cancer tumorigenesis.

## Methods

### Data and preprocessing

The processed exome-seq mutation data and clinical information for all patients in this study were downloaded from TCGA (“Level 2” data designation). We filtered out variants located in the 1000 Genome Project (Phase 3) [13], and the NHLBI GO Exome Sequencing Project (version 2) [14], which represented more than 200,000 individuals’ variants, and the Exome Aggregation Consortium (version 0.2) [15], which spanned 60,706 unrelated individuals’ variants by applying a minor allele frequency threshold of 0.1 to all three databases. Variants were annotated with the variant effector predictor tool [16]. Only the nonsynonymous mutations then remained. The annotated genes harboring each of nonsynonymous mutations were remained as mutated genes. All further analyses were based on these nonsynonymous mutations and mutated genes.

Pearson correlation was used to assess the correlation between the number of somatic and nonsynonymous mutations in each clinical stage. The difference in the number of nonsynonymous mutations in early stages (I and II) and later stages (III and IV) was performed by two-sided Student’s *t* test. *P* value less than 0.05 was considered to indicate statistical significance.

### Driver genes and core pathways collection

The 138 driver genes and 12 core cancer pathways were obtained from a previous report [17]. In general, a total of 18,306 mutated genes harboring 404,863 subtle mutations from the Catalogue of Somatic Mutations in Cancer (COSMIC) database [18] were assessed as driver genes, and it was determined whether each gene was likely to be an oncogene or tumor suppressor gene. The gene was classified as an oncogene if >20% of the recorded mutations in the gene occurred at recurrent positions and were missense. The gene was categorized as a tumor suppressor gene if >20% of the recorded mutations in the gene were inactivating. This “20/20 rule” [17] was applied to the selection of the 138 driver genes. These driver genes were classified into 12 signaling pathways regulating three core cellular processes: cell fate, cell survival, and genome maintenance [17].

### Pathway enrichment analysis

To quantify the association between the mutated genes in cervical cancer and the 12 core pathways, we downloaded the cancer-associated genes from the COSMIC database. Then, for each stage, we calculated the total number of non-redundant genes in COSMIC, the total number of non-redundant mutated genes in the corresponding stage, the number of genes in COSMIC that was found in each core pathway, and the number of mutated genes in each stage that was also found in each core pathway. The representation of each of the core pathway genes within the mutated genes in each stage was compared to the representation of those within all of the genes in the COSMIC database using Fisher’s exact test, similar to a previous approach [19]. The adjusted *p* value for each pathway was calculated using the Benjamini and Hochberg method. The significantly enriched pathway was considered if the adjusted *p* value was below 0.025.

### Post-translational modifications prediction

The missense mutations of all of the epigenetic regulators were extracted from the output of the variant effector predictor. For each epigenetic regulator, the mutant amino acid sequence was extracted from the corresponding wild-type amino acid sequence, with the mutated position substituted with the mutated residue. The paired wild-type sequence and the mutant sequence for each gene were constructed in a Fasta format. The ReKINect tool [20] was used to predict the likely functionality for each mutation.

The protein domains were derived from Uniprot. IBS software [21] was used to illustrate the protein domain structure and the amino acid changes.

### Immunogenic variants prediction

For each somatic point mutation, we obtained the corresponding mutated amino acid and constructed one peptide centered on the mutated residue, which was flanked on each side by eight amino acids from the protein sequence. We also obtained the corresponding normal 17-amino-acid peptide. The NETMHC-3.4 algorithm [22] was used to predict the binding affinity for the mutant peptide and the normal peptide with MHC-I, separately. For each mutant or normal 17-amino acid peptide, a peptide of length 9 was used to predict its binding affinity with MHC-I. The mutation exhibited immunogenicity only if the mutant peptide showed a strong binding affinity with MHC-I (affinity value <50) and the normal peptide had no binding affinity (affinity value >500) at the same peptide position.

### Mutational score calculation

Based on the nonsynonymous mutations of patients harboring *MLL2* mutations, we predicted the deleterious effects for each mutation in each patient by calculating a mutational score. Using a previously published strategy [23], each mutation was assigned a score that quantified its deleterious impact by integrating multiple factors, such as functional genomic data, transcription factor binding, transcript information, and protein level information. In general, a higher score indicates that a mutation is more likely to have deleterious effects. A gene’s mutational score was calculated by summing the scores of all the mutations found in the gene.

### Survival analysis

Survival curves were generated using the Kaplan-Meier method, and differences were evaluated using the Log-rank (Mantel-Cox) test. Overall survival was calculated from the time of initial diagnosis to death, or censored to the time when the patient was last known to be alive. *P* values under 0.05 were considered statistically significant. Hazard ratios and associated 95% confidence intervals were calculated with the Cox proportional-hazards model. All tests were two-sided and all calculations were performed with the R Version 3.1.1 statistical software.

## Results

### Patient data and somatic mutations

We collected exome-seq data for 194 cases. According to the International Federation of Gynecology and Obstetrics (FIGO) staging system [24], 121 patients had Stage I disease, 36 Stage II, 30 Stage III, and 7 Stage IV. After filtration, we obtained a total of 37,317 somatic mutations. We annotated these mutations, and 25,742 nonsynonymous mutations were used for downstream analyses. All of the mutated genes and nonsynonymous mutations are listed in Additional file 1: Table S1. We observed a strong correlation between the number of somatic and nonsynonymous mutations in each clinical stage (Additional file 2: Figure S1). Although we observed that the later stages (III and IV) exhibited higher incidences of nonsynonymous mutations than the early stages (I and II), the number of nonsynonymous mutations showed no significant correlation with the patients’ clinical stage (Student’s *t* test, *p* value: 0.4781; Additional file 3: Figure S2).

### The chromatin modification pathway is the most significantly altered

Of the approximately 20,000 protein-coding genes in the human genome, only 138 driver genes were reported and classified into 12 core pathways [17] (Additional file 4: Table S2). We researched whether these driver genes had been reported in another well-known cancer driver gene database, IntOgen [25]. Most of them (71%) had been detected as mutational cancer drivers (Additional file 4: Table S2). Using the 138 driver genes obtained from a previous report [17], we examined their mutations in our data. Of the 25,742 nonsynonymous mutations, a total of 105 driver genes of them harbored 491 mutations (Additional file 5: Table S3).

**Fig. 1:**
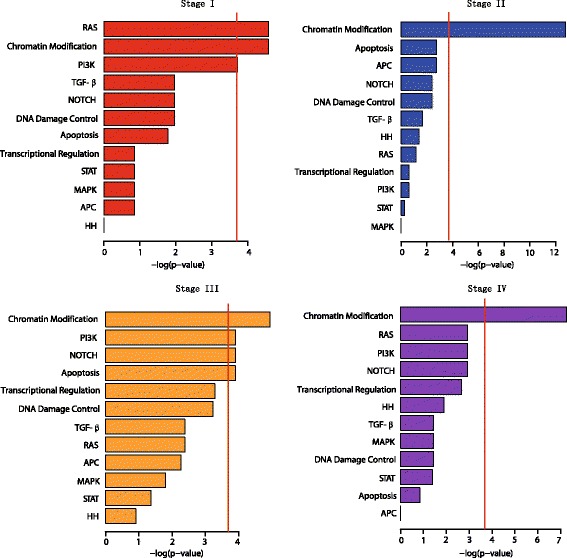
The chromatin modification pathway was the most significantly altered. *Bar plots* show the predefined core cancer pathways’ [17] enrichment of the mutated genes at each clinical stage. The bar plot in *red*, *blue*, *orange*, and *purple* represents Stage I, Stage II, Stage III, and Stage IV, respectively. The Fisher’s exact test was performed to calculate the *p* value. The adjusted *p* value for each pathway was calculated using the Benjamini and Hochberg method. The logarithm transformation (base 10) was applied to the adjusted *p* value. Vertical red line represents the cut-off of the adjusted *p* value of 0.025

Because these driver genes and core pathways play significant roles in tumorigenesis, we determined whether any of these pathways were significantly altered. All of the nonsynonymous mutations in all stages were integrated, and the pathway enrichment analysis revealed that the chromatin modification pathway was the most significantly altered across all stages, particularly at Stages II and IV (Fisher’s exact test, adjusted *p* value: 0.010344 (Stage I), adjusted *p* value: 2.8176e-6 (Stage II), adjusted *p* value: 7.1292e-3 (Stage III), adjusted *p* value: 7.0056e-4 (Stage IV); Fig. 1). At Stage I, the RAS (Fisher’s exact test, adjusted *p* value: 0.010344; Fig. 1) and PI3K (Fisher’s exact test, adjusted *p* value: 0.0245; Fig. 1) pathways were found to be significantly altered. The PI3K pathway was also significantly mutated at Stage III (Fisher’s exact test, adjusted *p* value: 0.020184; Fig. 1). The NOTCH (Fisher’s exact test, adjusted *p* value: 0.020184; Fig. 1) and apoptosis (Fisher’s exact test, adjusted *p* value: 0.020184; Fig. 1) pathways were also significantly altered at Stage III. Thus, the chromatin modification pathway dominated across all tumor stages.

**Fig. 2:**
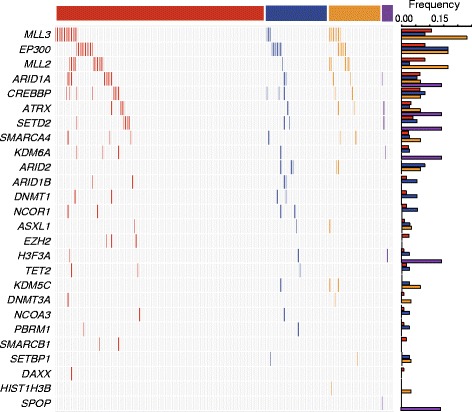
Mutated genes involved in the chromatin modification pathway. The heatmap shows the distribution of the mutated genes involved in the chromatin modification pathway across all clinical stages. Each column represents an individual, and each row denotes a gene. The *bar graph* on the top shows the Stage I, Stage II, Stage III, and Stage IV patient subgroups as *red*, *blue*, *orange*, and *purple*, respectively. The *bar plots* on the right side show the mutation frequency of each gene in each clinical stage colored by the stages

The mutated genes involved in these pathways are illustrated in Fig. 2 and Additional file 6: Figure S3. In the chromatin modification pathway, recurrent mutations in *MLL3*, *EP300*, *MLL2*, *ARID1A*, and *CREBBP* were present in 12%, 11%, 8%, 7%, and 7% of patients, respectively (Fig. 2). *EP300* was most recurrently mutated in the NOTCH pathway (11%), followed by *FBXW7* (9%) (Additional file 6: Figure S3). *NFE2L2* was recurrently mutated in the apoptosis pathway (6%) and *ERBB2* in the RAS pathway (5%) (Additional file 6: Figure S3). *PIK3CA* (9%), and *ERBB2* (5%) were recurrently mutated in the PI3K pathway (Additional file 6: Figure S3). Furthermore, *EP300*, *ARID1A*, *FBXW7*, *NFE2L2*, *PIK3CA,* and *ERBB2* have all been previously reported as pathogenic genes in cervical cancer [11, 12], and we highlighted the roles of *MLL2*, *MLL3*, and *CREBBP* in the tumorigenesis.

### Histone modification regulators are mainly mutated

**Fig. 3:**
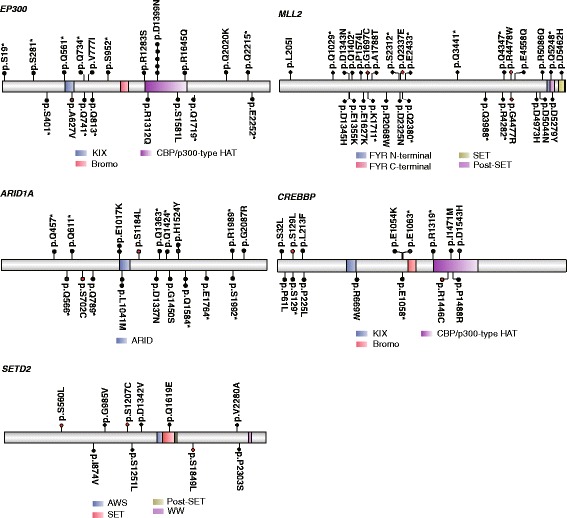
Recurrently mutated histone modification regulators harboring mutations destroying phosphorylation sites. *EP300*, *MLL2*, *ARID1A*, *CREBBP*, and *SETD2* are shown in the context of protein domain models derived from UniProt. Each filled circle represents a mutated patient. Somatic missense mutations destroying the phosphorylation sites are marked in red. Domains are depicted with various colors

After carefully observing the recurrently altered genes involved in the chromatin modification pathway (Fig. 2), it was clear that the histone modification regulators were primarily mutated. *MLL3* and *MLL2* both serve as enzymes in histone lysine methyl-transferation. Mutations in *MLL2* have been shown to be a cause of Kabuki syndrome [26]. One stop-gained mutation at residual Q5248 and one missense mutation at D5279 occurred in the FYR C-terminal domain, which is required to adopt an alpha + beta fold. Another missense mutation, p.D5462H, occurred in the SET domain that serves a function in lysine methyl-transferation (Fig. 3). The mutations occurring in these domains were different from those observed in Kabuki syndrome [26]. *EP300*, which was previously identified to be recurrently mutated in cervical cancer [11], has a high sequence similarity to *CREBBP*. Both were determined to harbor recurrent mutations in the CBP/p300-type HAT domain, which functions as a histone acetyl-transferation (Fig. 3). In *SETD2*, also a histone methyltransferase, a mutation p.Q1619E occurred at the SET domain, which also causes lysine methyl-transferation (Fig. 3). Thus, mutations occurring in these regulators may lead to abnormal enzyme activities, such as aberrant histone methylations.

Interestingly, of the 197 mutations in the 26 epigenetic regulators involved in the chromatin modification pathway, 25 missense mutations in 13 genes were predicted in or around the phosphorylation sites (Fig. 3; Additional file 7: Figure S4; Additional file 8: Table S4). Phosphorylation is an important post-translational modification that is capable of activating or inhibiting the activity of proteins in numerous biological processes [27, 28]. Mutations that destroy the phosphorylation sites may consequently disturb the signaling transduction network in the cell or affect their enzymatic activities.

### Mutations in *MLL2* are associated with worse survival

**Fig. 4:**
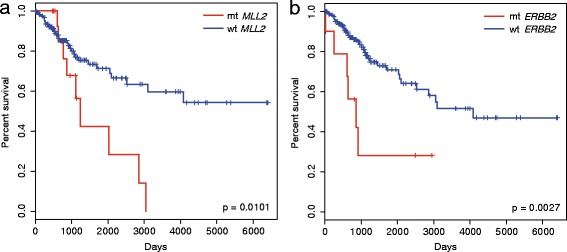
Overall survival of patients. Kaplan-Meier analysis of the survival of patients with and without *MLL2* (*n* = 194; 17 *MLL2* mt and 177 *MLL2* wt patients) (**a**), or *ERBB2* (*n* = 194; 10 *ERBB2* mt and 184 *ERBB2* wt patients) (**b**) mutations. Differences were evaluated by the Log-rank (Mantel-Cox) test. mt, mutant; wt, wild-type

We next ask whether these epigenetic regulator mutations affected patients’ survival. Of the mutated genes, patients with *MLL2* mutations exhibited a worse overall survival rate than those without (hazard ratio = 0.3912; 95% confidence interval, 0.1864 to 0.8212; *p* value: 0.0101; Fig. 4a). A risk of death reduction of around 61% was observed in those without *MLL2* mutations. No differences were observed in patients with and without either of the epigenetic regulatory gene mutations (Log-rank Mantel-Cox, *p* value: 0.131). Thus, *MLL2* may be a prognostic factor. We tested other recurrent genes in the PI3K, RAS, Apoptosis, and NOTCH pathways, including *PIK3CA*, *ERBB2*, *NFE2L2*, and *FBXW7*. Interestingly, though these genes were previously reported as pathogenic in cervical cancer, only patients harboring mutant *ERBB2* displayed poorer overall survival than those with wild-type (hazard ratio = 0.2864; 95% confidence interval, 0.12 to 0.6836; *p* value: 0.0027; Fig. 4b). *ERBB2* was involved in both the PI3K and RAS pathways. No differences were observed in patients with and without either of the mutations in these two pathways. Therefore, our data showed that, along with the previously reported pathogenic genes, mutations in *MLL2* also exhibited a new prognostic characteristic in cervical cancer.

### The impact of mutations in *MLL2*

**Fig. 5:**
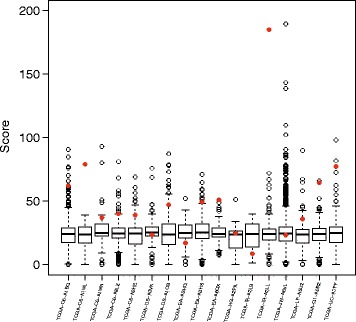
Mutational scores of *MLL2* mutations. For each nonsynonymous mutation in each patient harboring mutant *MLL2*, a mutational score was calculated as described previously [23]. A gene’s mutational score was calculated as the sum of all the scores of the mutations found in the gene. The *MLL2* (red) mutational score ranked higher than other variants

In addition, based on the mutation spectrum in the patients harboring *MLL2* mutations in our study, we predicted the impact of their mutations by quantifying the deleterious effects of the mutations. In general, this strategy scored each mutation by considering many factors, such as functional genomic data, transcription factor binding, transcript information, and protein level information [23], with a higher score indicating that a mutation is more likely to have deleterious effects. Among 17 patients in our data, we display here the scores of mutations in *MLL2* in 13 patients (76.5%), which is ranked in the top 10 mutated genes. In three patients, *MLL2* had the highest mutational score as compared to the majority of the other mutated genes in each patient (Fig. 5). It is highly probable that the mutations in *MLL2* could have deleterious effects to the human genome.

### The immunogenicity of mutations suggests potential immunotherapy

In malignant tumors harboring mutations, some of the mutations may generate “non-self” neo-antigens. These neo-epitopes, if presented to the cell surface through the binding with MHC-I which are also found on the cell surface, could be recognized by the immune system such as T lymphocytes. Those tumor cells could then be immunoreactive as an anti-tumor response could be triggered. The recent neo-antigen identification approach is based on somatic point mutation by predicting the binding affinity between the mutated peptide and MHC-I, which indicates that immunotherapy may be a promising new treatment [29–33].

**Fig. 6:**
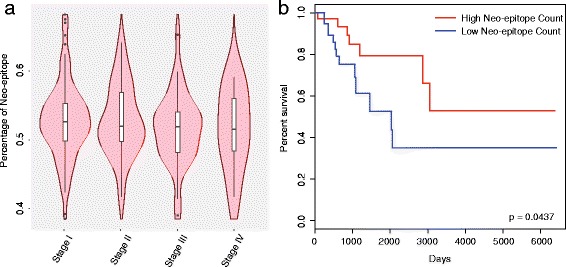
Percentage of neo-epitopes across stages and association with survival. **a** A violin plot shows the distribution of neo-epitopes percentage at each clinical stage. The neo-epitope percentage was calculated by dividing the total number of neo-epitopes by the total number of variants in each patient. **b** Overall survival for patients based on the neo-epitope number. Kaplan-Meier curves were constructed to look at the difference in the survival of patients with high and low numbers of neo-epitopes. Patients in the top 1/4 neo-epitope number were chosen as patients with high neo-epitope count, and the bottom 1/4 were chosen as the low neo-epitope count. Differences were evaluated by the Log-rank (Mantel-Cox) test (*n* = 95; 47 high neo-epitope count and 48 low neo-epitope count patients)

We examined all of the mutations’ immunogenicity by predicting their derived mutant peptides and their corresponding normal peptides’ binding affinities with MHC-I. On average, around 50% of the total variants’ peptides showed strong binding with MHC-I across all stages (Fig. 6a), while the corresponding normal peptides had no binding affinity at the same peptide position. This indicated that tumor cells in each stage can generate neo-epitopes that may induce an anti-tumor immune response. Moreover, we also observed marginal association between the number of neo-epitopes and patient survival (hazard ratio = 2.5185; 95% confidence interval, 0.9957 to 6.37; *p* value: 0.0437; Fig. 6b). Therefore, cervical cancer patients are likely to benefit from immunotherapy.

## Discussion

Papanicolaou smear and colposcopy programs can prevent cervical cancer development; however, 80% of diagnosed cases have already progressed to the later stages [34]. Women with cervical cancer remain a high-risk population for whom effective treatment options and reliable therapy targets are limited. In this study, we demonstrated that the chromatin modification pathways of cervical cancer patients were significantly altered. Mutations in *MLL2*, a histone methyltransferase, were associated with poor survival. This study indicates that genetic mutations in epigenetic regulators and potential epigenetic dysregulation play a role in the development of cervical cancer. As a result, our understanding of the pathogenesis of cervical cancer is greatly improved, and new therapeutic strategies are suggested.

Aberrant epigenetic changes in cervical cancer have been widely studied, and the main focus has been on DNA methylation [35–37], such as the hypermethylation of oncogenic genes [35]. In contrast, histone modification changes in cervical cancer have not been studied as extensively. Alterations of epigenetically modified genes were not examined in our study, but it was shown that epigenetic regulator genes were actually recurrently mutated in cervical cancer. It was also shown that they mainly had a histone methyl-transferation function. Thus, the mutation of these factors may consequently lead to abnormal histone modification in the genome. Interestingly, mutations in the histone methyltransferase *MLL2* that methylates the Lys-4 position of histone H3 exhibited worse overall survival. It is highly probable that the cervical cancer genome may harbor abnormal H3K4 methylation, which may shape a new epigenetic landscape that contributes to cancer deterioration. Approaches such as Chromatin immunoprecipitation (ChIP) followed by high-throughput DNA sequencing (ChIP-seq) for the H3K4me3, and other histone modifications in the patient tissues, deserve further study.

Half of the epigenetic regulators harbored mutations in or around the phosphorylation sites in their enzymes. Interestingly, the blocking of *EP300*’s phosphorylation was reported to decrease the proliferation and metastasis activity of lung cancer cells. The molecular mechanism showed that phosphorylation blocking in this protein disrupted chromatin condensation and increased the acetylation of histone H3 [38]. The phosphorylation of *MLL2*, controlled by CDK2, facilitated its recruitment to developmental genes in G1 in human pluripotent cells, consequently leading to changes in the developmental genes’ chromosome architecture [39]. Mutations occurring on the phosphorylation site in *CREBBP* were shown to result in inappropriate activation of gluconeogenesis [40]. The inhibition of *SETD2*’s phosphorylation by long non-coding RNA HOTAIR has been reported to trigger a reduction of trimethylation on histone H3 thirty-sixth lysine, consequently promoting human liver cancer stem cell malignant growth [41]. *SMARCA4*, also known as *BRG1*, was shown to modulate DNA double-strand break repair by its phosphorylation [42]. It has been suggested that the enzymatic activity of *DNMT1* is possibly modulated by phosphorylation [43], and it has been demonstrated that its phosphorylation by AKT1 kinase increases its stability and abundance [15]. The phosphorylation of *NCOR1* was shown to play a role in transcriptional regulation in prostate cancer [44] and in the liver in mice [45]. *EZH2*, despite of its ability to trimethylate lysine 27 in histone H3, when phosphorylated, suppressed its methyltransferase activity [46, 47], and switched to a coactivator for its oncogenic function in prostate cancer [48]. The phosphorylation of *DNMT3A* was found to be required for its localization to heterochromatin and capable of shaping the CpG methylome [49]. Phosphorylated *DAXX* was reported to facilitate DNA damage-induced *p53* activation [50]. Thus, it seems evident that phosphorylation is very important to these proteins’ normal functions. In some epigenetic regulators, phosphorylation is associated with cancer cell malignant growth. It will be interesting to explore the functional links between those specific phosphorylation events and the epigenetic regulators’ activities in cervical cancer.

Although the chromatin modification pathway was predominantly mutated across all clinical stages, others, such as the RAS, PI3K, NOTCH and apoptosis pathways, were also recurrently mutated at certain stages. These pathways and the recurrently mutated genes involved therein, such as *EP300*, *ARID1A*, *FBXW7*, *NFE2L2*, *PIK3CA*, and *ERBB2*, were all found to be pathogenic in previous cervical cancer studies [11, 12]. However, only mutations in *ERBB2* were associated with worse survival. *ERBB2* is involved in both the RAS and PI3K pathways. *MAP2K1* and *MAP3K1* were also mutated in the RAS pathway. Thus, the signal cascade, which should be activated when normally phosphorylated, may be disrupted. We observed that the mutations in some epigenetic regulators occurred around the phosphorylation sites. Currently, the temporal mutational order relationship or the association between these genetic events is unknown. The genetic mutations appear to have disrupted phosphorylation, which could together lead to a series of disorders in the cervical cancer cells.

One previous study of 115 cervical cancer samples from Norway and Mexico identified previously unknown somatic mutations that recurrently occurred in *EP300*, *FBXW7*, *NFE2L2*, *TP53*, and *ERBB2* [11]. Another study in 15 cervical cancer patients from Hong Kong revealed frequently altered genes, including *FAT1*, *ARID1A*, *ERBB2*, and *PIK3CA* [51]. One recent study of 228 cervical cancers using TCGA data identified *SHKBP1*, *ERBB3*, *CASP8*, *HLA-A*, and *TGFBR2* as novel significantly mutated genes, and previously identified pathogenic genes including *PIK3CA*, *EP300*, *ARID1A*, and *NFE2L2* were also confirmed [52]. Similarly, all of these genes were identified in each study using the same approach of analyzing the significantly mutated genes, whereas we only focused on the driver genes and core pathways that play significant roles in tumorigenesis. Some of the gene mutations reported in our study, such as *MLL3* and *MLL2*, were not previously identified in those studies, which may be because they did not satisfy the significance criteria. In contrast, other altered genes in this study, including *EP300*, *ARID1A*, *FBXW7*, *NFE2L2*, *PIK3CA,* and *ERBB2*, were reported as pathogenic genes in the aforementioned previous studies and were consistent with those cervical cancer genome studies. Interestingly, *CASP8*, which was newly identified as a significantly mutated gene in the recent study [52], was also included in the 138 driver genes in our study, and was frequently mutated in the apoptosis pathway which was significantly altered at Stage III (Fig. 1; Additional file 6: Figure S3). Additionally, unlike this study, other cervical cancer genome studies did not report the chromatin modification pathway as being predominately mutated as compared to other core cancer pathways. Among the genes involved in this pathway, we found that the mutations in *MLL2* were associated with poor survival in cervical cancer. The role of epigenetic regulator mutations has been identified as increasingly important in other cancers’ tumorigenesis [19, 53]; however, it has not been extensively explored in cervical cancer. This study is the first to highlight mutations in the epigenetic regulators, particularly the emerging role of *MLL2* in cervical carcinogenesis. Our results shed more light on the epigenetic mechanism underlying cervical cancer pathogenesis.

Most epigenetic therapy agents used in treatment are analogue inhibitors [35]. Clinical studies, however, have demonstrated their limitations, such as poor activity against solid tumors and toxicity [35, 54]. Thus, the merits of this targeted therapy have not yet been established. Accordingly, it is imperative that possible therapy strategies be identified, and the evidence increasingly suggests that T cells can provide clinical responses by recognizing unique neo-antigens expressed by somatic mutations in tumors [55–57]. By screening tumor-specific neo-antigens and identifying mutation-specific T cells, the immune targeting of cancer mutations has demonstrated therapeutic potential [30, 58]. The numerous neo-epitopes in our data derived from the mutations indicate that an anti-mutation T cell response might be feasible. Further investigation into potential immunotherapies for cervical cancer is warranted.

## Conclusions

The role of epigenetic regulator mutations in the tumorigenesis of other cancers has been recently highlighted [19, 53]. The association, however, between mutated epigenetic regulators and cervical carcinogenesis has not been extensively explored. To our knowledge, this is the first report to focus on mutations in the epigenetic regulators, particularly the central role of *MLL2* in the pathogenesis of cervical cancer on a genome-wide scale. Our results are important both for understanding cervical cancer development and for the continued search for possible therapy strategies.

## Additional files

Additional file 1: Table S1. All mutated genes and nonsynonymous mutations. (XLS 3318 kb)

Additional file 2: Figure S1. Correlation between the somatic mutations and nonsynonymous mutations. For each clinical stage, the number of total somatic mutations and the nonsynonymous mutations in each patient were plotted. The correlation coefficient and the significant *p* value are shown. (PDF 148 kb)

Additional file 3: Figure S2. Correlation between the nonsynonymous mutations with patients’ clinical stage. Distribution of patients’ nonsynonymous mutations number with the patients’ disease stages is exhibited. *P* value was calculated by a two-sided Student’s *t* test (mean ± s.d.; *n* = 194 subjects). (EPS 487 kb)

Additional file 4: Table S2. Driver genes and core pathways. (XLS 33 kb)

Additional file 5: Table S3. Mutated driver genes and variants in patients. (XLS 99 kb)

Additional file 6: Figure S3. Mutated genes involved in the PI3K, RAS, apoptosis, and NOTCH pathways. The heatmaps show the distribution of the mutated genes involved in the four pathways across all clinical stages. Each column represents an individual, and each row denotes a gene. The bar graph on the top shows the patients’ subgroup at Stage I, Stage II, Stage III, and Stage IV as red, blue, orange, and purple, respectively. (EPS 2420 kb)

Additional file 7: Figure S4. Another 8 epigenetic regulators harboring mutations destroying phosphorylation sites. The 8 epigenetic regulators are shown in the context of protein domain models derived from UniProt. Each filled circle represents a mutated patient. Somatic mutations destroying the phosphorylation sites are marked in red. Domains are depicted with various colors. (EPS 1080 kb)

Additional file 8: Table S4. Details of the 25 missense mutations destroying phosphorylation sites. (XLS 26 kb)

## Abbreviations

ChIP-seq: Chromatin immunoprecipitation (ChIP) followed by high-throughput DNA sequencing
COSMIC: The Catalogue of Somatic Mutations in Cancer
FIGO: The International Federation of Gynecology and Obstetrics staging system
HPV: Human Papilloma Virus
MHC-I: The major histocompatibility complex I molecules
TCGA: The Cancer Genome Atlas
