# Supplementary figures and images for: Emerging role of mutations in epigenetic regulators including MLL2 derived from The Cancer Genome Atlas for cervical cancer
Source: BMC Cancer. 2017 Apr 8;17:252. doi: 10.1186/s12885-017-3257-x (PMC5385072; doi:10.1186/s12885-017-3257-x)

Stage I

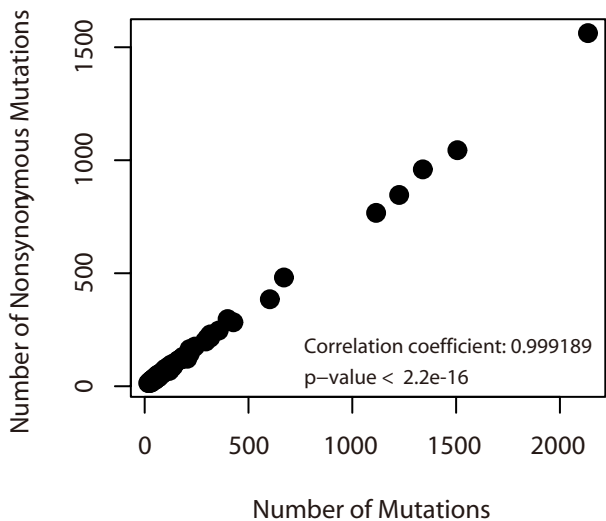

Supplement: Supplementary file 2 — Correlation between the somatic mutations and nonsynonymous mutations. For each clinical stage, the number of total somatic mutations and the nonsynonymous mutations in each patient were plotted. The correlation coefficient and the significant p value are shown. (PDF 148 kb) [file 12885_2017_3257_MOESM2_ESM.pdf]
